# Targeting of BMI-1 with PTC-209 shows potent anti-myeloma activity and impairs the tumour microenvironment

**DOI:** 10.1186/s13045-016-0247-4

**Published:** 2016-03-02

**Authors:** Arnold Bolomsky, Karin Schlangen, Wolfgang Schreiner, Niklas Zojer, Heinz Ludwig

**Affiliations:** Wilhelminen Cancer Research Institute, Department of Medicine I, Wilhelminenspital, Montleartstraße 37, 1160 Vienna, Austria; Center for Medical Statistics, Informatics and Intelligent Systems, Medical University of Vienna, Vienna, Austria

**Keywords:** Multiple myeloma, BMI-1, PTC-209, Microenvironment

## Abstract

**Background:**

The polycomb complex protein BMI-1 (BMI-1) is a putative oncogene reported to be overexpressed in multiple myeloma (MM). Silencing of BMI-1 was shown to impair the growth and survival of MM cells. However, therapeutic agents specifically targeting BMI-1 were not available so far. Here, we investigated PTC-209, a novel small molecule inhibitor of BMI-1, for its activity in MM.

**Methods:**

*BMI-1* expression was analysed in human MM cell lines and primary MM cells by using publically available gene expression profiling (GEP) data. The anti-MM activity of PTC-209 was investigated by viability testing, cell cycle analysis, annexin V and 7-AAD staining, quantification of cleaved poly(ADP-ribose) polymerase (PARP), JC-1 as well as colony formation assays. Deregulation of central myeloma growth and survival genes was studied by quantitative PCR and flow cytometry, respectively. In addition, the impact of PTC-209 on in vitro osteoclast, osteoblast and tube formation was analysed.

**Results:**

We confirmed overexpression of *BMI-1* in MM patients by using publically available GEP datasets. Of note, *BMI-1* expression was further increased at relapse which translated into significantly shorter overall survival in relapsed/refractory patients treated with bortezomib or dexamethasone.

Treatment with PTC-209 significantly decreased viable cell numbers in human MM cell lines, induced a G1 cell cycle arrest, promoted apoptosis and demonstrated synergistic activity with pomalidomide and carfilzomib. The anti-MM activity of PTC-209 was accompanied by a significant decrease of cyclin D1 (*CCND1*) and v-myc avian myelocytomatosis viral oncogene homolog (*MYC*) expression as well as upregulation of cyclin-dependent kinase inhibitor 1A (*CDKN1A*) and cyclin-dependent kinase inhibitor 1B (*CDKN1B*). We also observed upregulation of *NOXA* (up to 3.6 ± 1.2-fold induction, *P* = 0.009) and subsequent downregulation of myeloid cell leukemia 1 (MCL-1) protein levels, which likely mediates the apoptotic effects of PTC-209. Importantly, the anti-MM activity was upheld in the presence of stromal support or myeloma growth factors insulin-like growth factor 1 (IGF-1) and interleukin 6 (IL-6).

In the MM microenvironment, PTC-209 impaired tube formation, impaired osteoclast development and decreased osteoblast formation in a dose-dependent manner (*P* < 0.01 at 1 μM, respectively). The latter might be attributed to an induction of DKK1 and was reversed by concurrent anti-DKK1 antibody treatment.

**Conclusions:**

We confirmed overexpression of *BMI-1* in MM highlighting its role as an attractive drug target and reveal therapeutic targeting of BMI-1 by PTC-209 as a promising novel therapeutic intervention for MM.

**Electronic supplementary material:**

The online version of this article (doi:10.1186/s13045-016-0247-4) contains supplementary material, which is available to authorized users.

## Background

Multiple myeloma (MM) arises from the clonal growth of malignant plasma cells in the bone marrow (BM) [1]. Treatment options for MM are continuously improving, leading to significantly increased response rates as well as prolonged survival [1, 2]. Despite this progress, myeloma remains a difficult-to-treat disease with the vast majority of patients eventually relapsing. Therefore, the identification of novel drug targets and introduction of additional therapeutic agents are urgently needed to improve the efficacy of existing therapies, to overcome drug resistance and to unravel additional drugable key players in the pathophysiology of MM.

The polycomb complex protein BMI-1 (BMI-1) constitutes a pleiotropic factor with implications in the regulation of the cell cycle, DNA damage response, apoptosis, senescence as well as stem cell self-renewal and differentiation [3]. BMI-1 was originally discovered as a cooperation factor for v-myc avian myelocytomatosis viral oncogene homolog (MYC) in lymphomagenesis and constitutes a central component of the polycomb repressive complex 1 (PRC1), an epigenetic repressor complex which acts through histone H2A mono-ubiquitination at lysine 119 [4–8]. Overexpression of BMI-1 was frequently observed in diverse human malignancies and associated with tumour initiation and propagation, disease progression and poor prognosis [9–13]. Moreover, BMI-1 was shown to mediate the growth and survival of cancer stem cells in several solid and haematological malignancies [14–17].

BMI-1 represents an attractive drug target in myeloma as well. Upregulation of BMI-1 has been reported previously in MM, and silencing of BMI-1 by small hairpin (sh) RNA significantly impaired the proliferation and colony formation of myeloma cells [18, 19]. Furthermore, silencing of BMI-1 induced apoptosis in vitro and in vivo through upregulation of BCL2-like 11 (*Bim*) expression in MM cells [19]. More recent results demonstrated that shRNA-mediated silencing of BMI-1 also sensitizes myeloma cells to bortezomib, which was attributed to increased expression of p21 and BCL2-associated X protein (*Bax*) [20]. However, despite the identification of BMI-1 as an attractive drug target in myeloma and various other malignancies, inhibitors specifically targeting BMI-1 have not been available so far.

Kreso et al. recently reported targeting of colorectal carcinoma with PTC-209, a novel small molecule inhibitor of BMI-1 [21]. Treatment of colorectal cancer cells reduced BMI-1 protein levels and significantly impaired tumour growth in vitro and in vivo. Importantly, PTC-209 also targeted cancer-initiating cells [21]. Treatment of chronic and acute myeloid leukemia cells with PTC-209 was likewise shown to impair tumour growth and survival [22, 23]. We therefore aimed to investigate the pre-clinical activity of PTC-209 in myeloma and to explore the impact of BMI-1 inhibition on the tumour microenvironment.

## Results

### BMI-1 is overexpressed in multiple myeloma and associated with survival

**Fig. 1:**
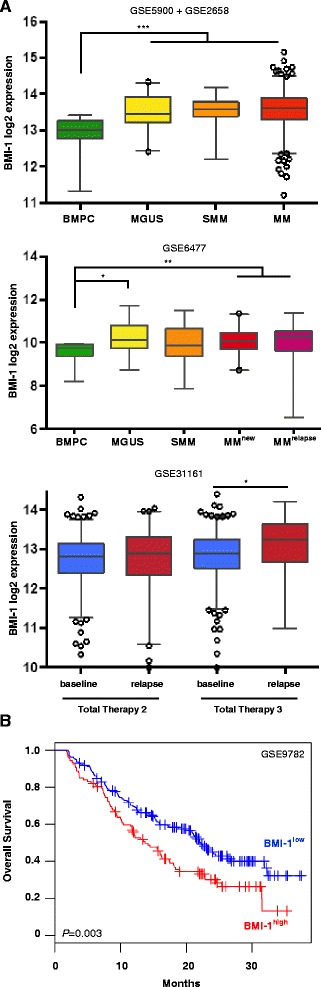
BMI-1 is overexpressed in multiple myeloma and associated with outcome. **a** *BMI-1* expression analysis of CD138^+^ purified cells in publically available gene expression datasets displayed significant overexpression in MGUS, SMM and MM patients compared to healthy donor plasma cells. In addition, *BMI-1* expression was increased at relapse (*n* = 29) compared to baseline (*n* = 433) in patients treated within TT3, but not in those of TT2 (*n* = 172 and *n* = 346, respectively). *Boxplots* represent median *BMI-1* expression (*line*) and 2.5–97.5 percentile (*bars*). ***P < 0.001, **P < 0.01 and *P < 0.05. **b** High *BMI-1* expression was associated with poor outcome in relapsed and/or refractory patients treated with bortezomib or dexamethasone (GSE9782) (*n* = 264). Samples were divided into two groups based on the maximally selected rank statistics cutoff

We confirmed overexpression of *BMI-1* in CD138^+^ purified cells of monoclonal gammopathy of undetermined significance (MGUS), smouldering multiple myeloma (SMM), newly diagnosed and relapsed MM patients compared to healthy controls in publically available gene expression profiling (GEP) datasets. As expected, *BMI-1* expression was significantly (*P* < 0.0001) elevated in MM patients (newly diagnosed and relapsed) compared to healthy donor bone marrow plasma cells (BMPCs). Of note, *BMI-1* expression was already increased in CD138^+^ cells of MGUS and SMM patients. We also examined *BMI-1* expression levels in total therapy 2 (TT2)- and TT3-treated patients at baseline and relapse. This analysis indeed demonstrated a significant increase of *BMI-1* expression at relapse in patients treated within the TT3 protocol (*P* = 0.016) (Fig. 1a). In line with this observation, relapsed and/or refractory patients with low *BMI-1* expression treated with bortezomib or dexamethasone displayed a superior prognosis compared to patients with high *BMI-1* expression (median overall survival [OS] 22.2 vs 13.7 months, *P* = 0.003) (Fig. 1b). These results confirmed overexpression of *BMI-1* in all stages of MM progression and therefore highlight its putative role as an attractive drug target in myeloma.

### PTC-209 impairs myeloma cell growth and survival

**Fig. 2:**
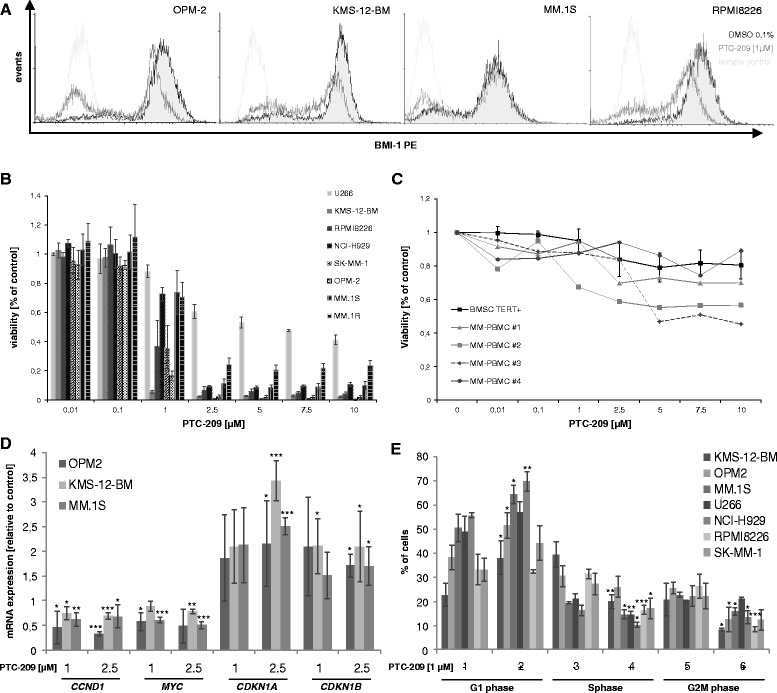
PTC-209 impairs myeloma cell viability and proliferation. **a** PTC-209 downregulated BMI-1 protein levels in all MM cell lines analysed by intracellular staining using flow cytometry. *Histograms* are representative for three independent experiments. **b** Reduced viability 96 h post treatment was observed in all MM cell lines in a dose-dependent manner, but not in BMSCs or PBMCs (**c**). **d** Downregulation of *CCND1* and *MYC* as well as upregulation of *CDNK1A* and *CDKN1B* was noted 5 h post treatment. **e** Deregulation of proliferation-associated genes translated into a significant increase of cells in the G1 phase and concurrent decrease in the S and G2M phase of the cell cycle 24 h post treatment. ***P < 0.001, **P < 0.01 and *P < 0.05 vs DMSO control

In line with the GEP analysis and previous reports, BMI-1 gene and protein expression was observed in eight of eight human myeloma cell lines (HMCLs) tested (not shown). Treatment with PTC-209 led to downregulation of BMI-1 protein levels (Fig. 2a) and significantly impaired viability of all HMCLs analysed with IC50 values <2 μM in six of eight HMCLs (range 0.21–5.68 μM) (Fig. 2b). No significant association was observed between IC50 values and BMI-1 mRNA (*R* =−0.32, *P* = 0.44) or protein expression (*R* =−0.43, *P* = 0.29). In contrast to HMCLs, IC50 values were not reached in healthy donor peripheral blood mononuclear cells (PBMCs) (range 15–629 μM) and bone marrow stromal cells (BMSCs) TERT^+^ cells (IC50: 54 μM) (Fig. 2c). Analysis of gene expression after short-term incubation (5 h) with PTC-209 demonstrated deregulation of cell cycle-associated genes. We observed significant downregulation of cyclin D1 (*CCND1*) (up to 0.67 ± 0.04-fold reduction, *P* < 0.001) and *MYC* (up to 0.50 ± 0.07-fold reduction, *P* < 0.001) as well as upregulation of the cell cycle inhibitory genes cyclin-dependent kinase inhibitor 1A (*CDKN1A*) (up to 3.4 ± 0.4-fold increase, *P* < 0.001) and cyclin-dependent kinase inhibitor 1B (*CDKN1B*) (up to 2.1 ± 0.6-fold increase, *P* = 0.03) (Fig. 2d). This translated into a significant accumulation of cells in the G1 phase and concurrent reduction of cells in the S and G2M phase of the cell cycle after 24 h of treatment with PTC-209 at 1 μM (Fig. 2e).

**Fig. 3:**
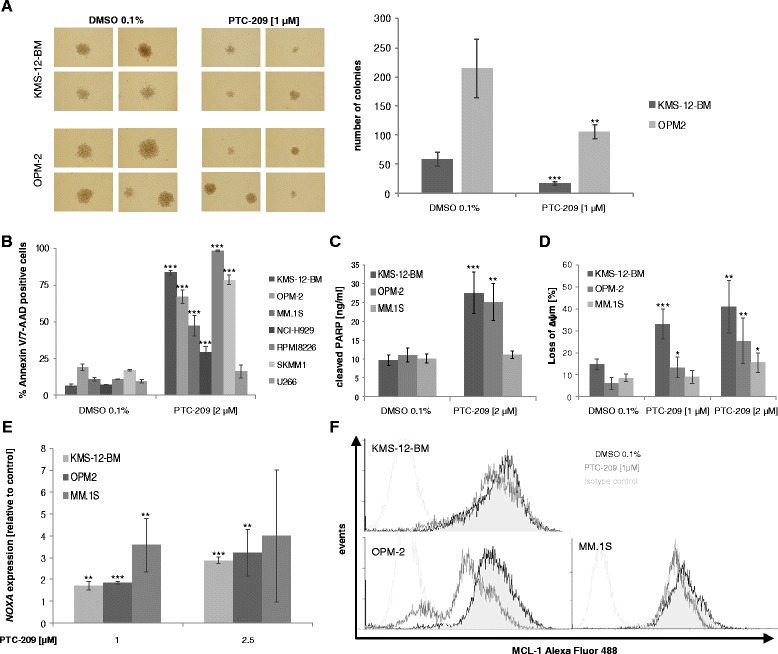
PTC-209 inhibits colony formation and induces apoptosis in myeloma cells. **a** Treatment with PTC-209 significantly inhibited colony formation of KMS-12-BM and OPM-2 cells. *Images* are representative for three independent experiments. Induction of apoptosis was verified by annexin V/7-AAD staining (**b**), quantification of cleaved PARP by ELISA (**c**) and analysis of the mitochondrial membrane potential by JC-1 assay (**d**) 72 and 24 h post PTC-209 treatment, respectively. **e** Rapid induction (5 h) of *NOXA* expression was observed in all MM cell lines tested, followed by a decrease in MCL-1 protein levels 20 h post treatment analysed by intracellular staining using flow cytometry (**f**). *Histograms* are representative for three independent experiments. ***P < 0.001, **P < 0.01 and *P < 0.05 vs DMSO control

In addition to the anti-proliferative effects, PTC-209 significantly impaired the number and size of colonies formed by myeloma cells in a colony formation assay (OPM-2: 215 ± 50 vs 105 ± 12 colonies with PTC-209 at 1 μM, *P* = 0.005; KMS-12-BM: 59 ± 12 vs 17 ± 3, *P* < 0.001) and induced apoptosis in all HMCLs analysed (Fig. 3a, b). The latter was further confirmed by the presence of increased poly(ADP-ribose) polymerase (PARP) cleavage and JC-1 assay, which indicated depolarization of the mitochondrial membrane after 24 h treatment with PTC-209 (Fig. 3c, d). Of note, viability 96 h post treatment with PTC-209 at 1 μM significantly correlated with the number of apoptotic cells at 72 h post treatment (*R* =−0.78, *P* = 0.04), but not with changes in the cell cycle profile. This suggests that induction of apoptosis is the main mechanism responsible for the reduction of viable cells upon PTC-209 treatment. We therefore assessed the regulation of mitochondrial genes associated with apoptosis and detected significant induction of *NOXA* expression in the presence of PTC-209 (up to 3.6 ± 1.2-fold increase, *P* = 0.009) (Fig. 3e). In contrast, no impact of PTC-209 was observed on *Bim* and *Bax* expression levels (data not shown). In line with the proposed functions of NOXA, we observed downregulation of myeloid cell leukemia 1 (MCL-1) protein levels (Fig. 3f), suggesting that induction of apoptosis by PTC-209 is related to NOXA-mediated inhibition of MCL-1.

### PTC-209 impairs the activity of stromal support for myeloma cells and shows synergistic activity with pomalidomide and carfilzomib

**Fig. 4:**
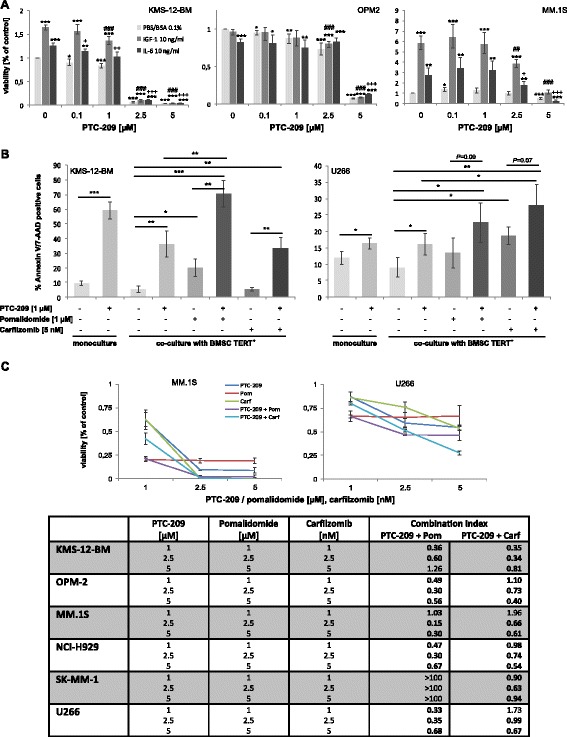
PTC-209 reduces the effect of major myeloma growth factors and stromal support as well as displays synergistic activity with pomalidomide and carfilzomib. **a** PTC-209 was found to impair the growth and survival-propagating effects of IGF-1 and IL-6 in a dose-dependent manner. **b** The anti-MM activity of PTC-209 was upheld in the presence of BM stromal cells and promoted the activity of pomalidomide and carfilzomib. ***P < 0.001, **P < 0.01 and *P < 0.05 vs PBS control; ^###^ *P* < 0.001, ^##^ *P* < 0.01 and ^#^ *P* < 0.05 vs IGF-1 control; ^+++^ *P* < 0.001, ^++^ *P* < 0.01 and ^+^ *P* < 0.05 vs IL-6 control. **c** Additive/synergistic activity of drug combinations was confirmed by concurrent treatment of MM cell lines with PTC-209 and either pomalidomide or carfilzomib for 96 h at varying concentrations. *Graphs* for MM.1S and U266 are representative for the panel of HMCLs analysed. Combination index (CI) values were determined with CompuSyn. *CI values* <0.8, 0.8–1.2, or >1.2 indicate synergistic, additive or antagonistic drug activities, respectively

To assess whether PTC-209 overcomes stromal-mediated drug resistance, we tested the activity of PTC-209 in the presence of insulin-like growth factor 1 (IGF-1) and interleukin 6 (IL-6). Importantly, PTC-209 was found to impair the growth- and survival-propagating effects of both soluble factors in a dose-dependent manner in the non-autonomously surviving cell lines KMS-12-BM and MM.1S. In the autonomously surviving cell line OPM-2 (proliferate in serum-free Syn-H medium), IGF-1 and IL-6 did not show any additional effect but likewise did not rescue OPM-2 cells from the anti-MM activity of PTC-209 (Fig. 4a). When KMS-12-BM and U266 cells were co-cultured with human BMSCs, PTC-209 significantly increased the rate of apoptotic cells (KMS-12-BM: 5.4 vs 36.1 % apoptotic cells with PTC-209 at 1 μM, *P* = 0.004; U266: 8.9 vs 16.0 %, *P* = 0.05). Moreover, PTC-209 enhanced the anti-MM activity of pomalidomide and carfilzomib in the presence of BMSCs; although, we have to note that there was only a marginal effect in the partial resistant HMCL U266 (Fig. 4b). To verify the synergistic activity of these agents, six HMCLs were incubated with PTC-209 and either pomalidomide or carfilzomib at varying concentrations. PTC-209 displayed synergistic and/or additive drug activity in combination with carfilzomib in all HMCLs analysed, and in five of six HMCLs in combination with pomalidomide. Importantly, synergistic activity with carfilzomib and pomalidomide was also observed in the PTC-209 partly resistant HMCL U266 (Fig. 4c). Similar results were obtained when PTC-209 was combined with dexamethasone (Additional file 1: Figure S1).

### PTC-209 targets the myeloma microenvironment

**Fig. 5:**
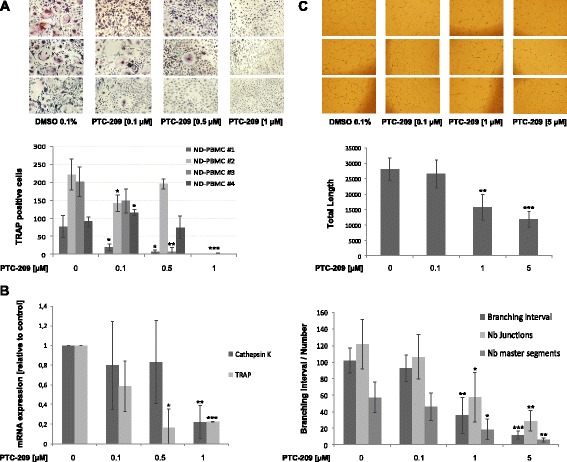
PTC-209 impairs in vitro osteoclast and tube formation. **a** PTC-209 significantly inhibited osteoclast formation in a dose-dependent manner verified by reduced numbers of multinucleated TRAP-positive cells at day 14 of differentiation. **b** The inhibitory impact on osteoclast formation was confirmed by decreased expression of cathepsin K and TRAP. **c** Tube formation was inhibited by PTC-209 in a dose-dependent manner. Analysis with the *Angiogenesis Analyzer for ImageJ* demonstrated a significant impact of PTC-209 on the total length, the number of junctions and master segments as well as the branching interval (defined as total segments length/number of branches) during the tube formation process. *Images* are representative for three independent experiments. ***P < 0.001, **P < 0.01 and *P < 0.05 vs DMSO control

As PTC-209 was shown to impair stromal-mediated drug resistance, we were interested whether it impacts the function of other cells in the myeloma microenvironment as well. Enhanced formation of osteoclasts and angiogenesis is a major hallmark of myeloma. We therefore analysed the activity of PTC-209 on these cell types. In vitro osteoclast formation of healthy donor PBMCs was significantly impaired, with no signs of tartrate-resistant acid phosphatase (TRAP)-positive osteoclasts when PTC-209 was used at 1 μM (Fig. 5a). This was further confirmed by decreased expression of cathepsin K and TRAP (0.88 ± 0.17 and 0.78 ± 0.01-fold downregulation with 1 μM PTC-209; *P* < 0.01, respectively) at day 14 of osteoclast formation (Fig. 5b). Similarly, PTC-209 was found to impair in vitro tube formation in a dose-dependent manner. Treatment with PTC-209 at 1 μM significantly decreased the total length (*P* = 0.005), the branching interval (*P* = 0.002) as well as the number of junctions and master segments (*P* = 0.02 and *P* = 0.01, respectively) of tubes formed by human umbilical vein endothelial cells (HUVECs) after a 19-h incubation period (Fig. 5c).

**Fig. 6:**
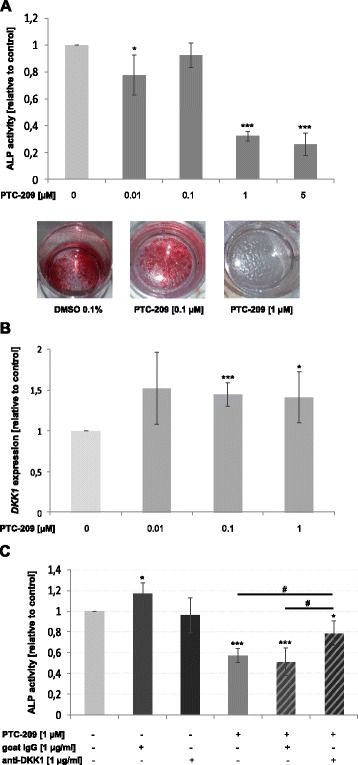
PTC-209 inhibits osteogenesis via upregulation of DKK1. **a** PTC-209 significantly inhibited osteoblast formation in a dose-dependent manner verified by reduced alkaline phosphatase activity and matrix mineralization at days 14 and 21 of differentiation, respectively. *Images* are representative for three independent experiments. **b** Treatment with PTC-209 increased *DKK1* expression in developing osteoblasts at day 14 of osteogenesis. **c** The inhibitory effect of PTC-209 on osteoblast activity was partially overcome by concurrent anti-DKK1 antibody treatment. ***P < 0.001, **P < 0.01 and *P < 0.05 vs DMSO control; ^#^ *P* < 0.05

As BMI-1 is known for its close interaction with the Wnt signalling pathway, we speculated that PTC-209 might affect osteoblast formation as well. We indeed observed a significant negative impact of PTC-209 on osteogenesis, evidenced by decreased alkaline phosphatase (ALP) activity (68 ± 4 % reduction with PTC209 at 1 μM, *P* < 0.001) and matrix mineralization in a dose-dependent manner (Fig. 6a). The reduction in osteoblast formation was accompanied by a significant increase in Dickkopf-1 (*DKK1)* expression at day 7 of osteogenesis (1.5 ± 0.1-fold increase at 0.1 μM PTC-209, *P* < 0.001) (Fig. 6b). We therefore investigated whether concurrent blockade of DKK1 with a neutralizing antibody could overcome the inhibitory effects on osteoblast formation. Bi-weekly treatment with anti-DKK1 antibody significantly increased ALP activity in the presence of PTC-209 at 1 μM (43 ± 6 vs 21 ± 12 % decrease in ALP activity, *P* = 0.02), suggesting that the osteoblast inhibitory properties of PTC-209 might be, at least in part, mediated by DKK1 (Fig. 6c).

## Discussion

In spite of the recent advances in the treatment of MM, the recurrence of myeloma after response to existing therapies is a major drawback on the way to cure. The identification of novel therapy targets and subsequent implementation of new anti-myeloma therapeutics is therefore urgently needed. Based on previous reports, inhibition of the polycomb complex protein BMI-1 might represent an attractive treatment approach for myeloma [19, 20], but therapeutic agents targeting BMI-1 are not available for clinical use so far. In the current study, we investigated the anti-MM activity of PTC-209, a novel small molecule inhibitor of BMI-1.

Our initial analysis of publically available GEP datasets confirmed the overexpression of *BMI-1* in MM. Overexpression of *BMI-1* has been reported in various malignancies, including MM [18], and is typically associated with poor survival [9–13]. We likewise observed a significant elevated expression of *BMI-1* in MM as well as in MGUS and SMM patients. Of note, *BMI-1* expression was further elevated in relapsed TT3, but not TT2 patients. This suggests that the use of distinct treatment strategies such as the addition of bortezomib in TT3 specifically impacts BMI-1 levels. According to this assumption, shRNA-mediated silencing of BMI-1 was shown to sensitize MM cells to bortezomib [20]. Our observation of increased *BMI-1* expression in relapsed TT3 patients suggests that further BMI-1 upregulation might confer a more aggressive phenotype during the progression of MM as it was shown in the progression of several other tumour entities [9, 12, 24–28]. This is also evidenced by an association of high *BMI-1* expression with worse overall survival in relapsed and/or refractory patients treated with bortezomib or dexamethasone (Fig. 1b) [29]. These results confirmed *BMI-1* overexpression in all stages of MM, from the onset of the disease until progression in response to therapy, underlining its central role as an attractive drug target in MM.

PTC-209 demonstrated significant anti-myeloma activity in all HMCLs analysed. In line with the effect of shRNA-mediated silencing of BMI-1 [19], we observed a significant impact on the colony formation of myeloma cells, suggesting that targeting BMI-1 also affects the viability of tumour-propagating cells. Recent reports indicated that PTC-209 targets cancer-initiating cells in colorectal and biliary tract cancer. In particular, PTC-209 impaired sphere formation in both entities as well as growth of aldehyde dehydrogenase-positive (ALDH^+^) cells in certain biliary tract cancer cell lines [21, 30]. Future studies therefore have to clarify whether BMI-1 inhibition specifically targets tumour-propagating cells in MM as well.

Similar to shRNA-mediated BMI-1 inhibition in breast and lung adenocarcinoma cells [31, 32], the growth-inhibiting effect of PTC-209 was associated with deregulation of *CCND1*, *MYC*, *CDKN1A* and *CDKN1B*. These genes are known to be implicated in the proliferation of MM cells and their deregulation therefore likely explains the accumulation of cells in the G1 phase and the impaired entry into the S and G2M phase of the cell cycle. Induction of apoptosis was accompanied by a rapid increase of *NOXA* expression and subsequent reduction of MCL-1 protein levels. Prior studies reported that silencing of BMI-1 in MM cells was linked to increased expression of either Bim or Bax. However, in these studies, upregulation of Bim and Bax reached significance 48 h post BMI-1 silencing [19, 20]. In the current study, upregulation of *NOXA* (but not Bim or Bax) was already observed 5 h post treatment with PTC-209, suggesting that NOXA might be upstream of Bim and Bax in the initiation of apoptosis after impairing BMI-1. According to this assumption, upregulation of NOXA leads to increased binding of NOXA to MCL-1, thereby releasing Bim from MCL-1 which subsequently mediates Bax (and Bak)-dependent induction of apoptosis [33, 34]. Similar to our results, a time-dependent increase of NOXA prior to Bim protein levels was observed in chronic lymphatic leukemia cells in response to histone deacetylase inhibitors (HDACi). HDACi were shown to induce a rapid increase of *NOXA* mRNA levels, which subsequently triggers MCL-1 binding and induces apoptosis [35]. Moreover, BMI-1 was shown to mediate the survival of memory CD4 T cells as well as mantle cell lymphoma cells via direct binding to the *NOXA* gene locus and repression of NOXA mRNA expression through histone modifications [14, 36]. These findings suggest that early upregulation of *NOXA* might release and activate Bim and Bax to exert their apoptotic effects upon BMI-1 inhibition.

Importantly, the anti-MM activity of PTC-209 was upheld in the presence of major myeloma growth factors (IGF-1 and IL-6) as well as in co-culture with BMSCs. Moreover, we observed synergistic activity of PTC-209 with pomalidomide, carfilzomib and dexamethasone, suggesting that inhibition of BMI-1 might improve current treatment strategies. A similar observation was made when BMI-1-silenced myeloma cells were treated with bortezomib. Concurrent treatment enhanced the anti-proliferative and apoptotic activity of bortezomib via pronounced induction of p21 and Bax [20].

In addition to its direct anti-myeloma effect, we demonstrated that PTC-209 showed a significant impact on stromal compartments as well. Little is known about the role of BMI-1 in the fate of BM environmental cells. Low expression levels of BMI-1 were associated with senescence in endothelial cells of the human cornea [37]. Moreover, BMI-1 was shown to promote the angiogenic activity of glioma and hepatocellular carcinoma cells [38–40]. In the current study, PTC-209 significantly inhibited osteoclast and tube formation in vitro. Increased osteoclast activity and formation as well as induction of angiogenesis are prominent features of the myeloma microenvironment. The interaction of myeloma cells and these compartments is implicated in tumour growth, progression and drug resistance [41]. Interfering with these manifestations therefore impairs MM cell growth and survival. Treatment with PTC-209 might thus not only target MM via direct effects on tumour cells, but also by impairing the crosstalk between tumour and stromal cells.

BMSCs of BMI-1^−/−^ mice were shown to undergo a shift from osteogenesis to adipogenesis. Moreover, BMI-1^−/−^ mice displayed an osteopenic phenotype characterized by skeletal growth retardation, decreased osteoblast numbers and activity [42, 43]. We also observed reduced osteoblast activity and formation in the presence of PTC-209. Considering the negative regulation of osteoblast development by myeloma cells, blockade of BMI-1 could aggravate these effects probably leading to skeletal-related side effects. We therefore aimed to identify the underlying mechanism for the decreased osteoblast formation. As BMI-1 is known for its close interaction with the Wnt signalling pathway [31], a major signalling pathway in osteogenesis [44], we speculated that the osteoblast inhibition observed in our study might be related to this connection. We indeed revealed a significant induction of *DKK1* expression in developing osteoblasts during PTC-209 treatment and that blockade of DKK1 with a specific antibody, at least in part, reversed the suppressive effect of PTC-209 on osteoblast activity. This suggests that combination therapy with anti-DKK1 antibodies might overcome the osteoblast suppressive effects of BMI-1 inhibition. In line with our results, silencing of BMI-1 in breast cancer cells was shown to impair Wnt signalling via downregulation of Wnt ligands (e.g. Wnt3a) and upregulation of Wnt inhibitors including DKK1. BMI-1 knockout was shown to upregulate DKK1 and to target cancer cells via subsequent downregulation of *MYC* and *CCND1* [31]. Interestingly, short-term treatment (5 h) with PTC-209 was found to induce *DKK1* expression in myeloma cells as well (up to 5.0 ± 1.9-fold increase, *P* < 0.05) (data not shown). This assumes that targeting Wnt signalling via BMI-1 blockade might also target myeloma cells. In line with this, inhibition of the Wnt signalling pathway was recently shown to affect the survival of mantle cell lymphoma-initiating cells [45]. Considering the proposed roles of Wnt signalling in disease progression and therapy resistance [46–48], BMI-1 inhibition could significantly improveme existing therapies by overcoming drug resistance.

Numerous small molecule inhibitors are currently in clinical development to improve the treatment opportunities for MM patients. These include Bruton’s tyrosine kinase [49], mitogen-activated protein kinase (MAPK) signalling cascade [50], phosphoinositol-3 kinase/AKT [51] and Bcl-2 family inhibitors [52] among others. Future studies have to clarify which of these agents provide the most potent anti-tumour activity, define predictive markers for an individualized treatment approach and examine the additive value in combination with standard regimens. Targeting of BMI-1 represents a promising novel therapeutic strategy among these evolving arsenals of specific inhibitors due to its universal expression pattern in MM and its impact on the myeloma microenvironment. Further studies evaluating the role of BMI-1 inhibition in myeloma and the applicability of more selective inhibitors (e.g. PTC596) in vitro and in vivo are therefore warranted.

## Conclusions

We confirmed overexpression of *BMI-1* in MM highlighting its role as an attractive drug target. In line with this, PTC-209 demonstrates potent anti-MM activity by targeting central myeloma survival genes (e.g. *MYC*, *MCL-1*), shows synergistic activity with pomalidomide, carfilzomib and dexamethasone, reduces the protective effect of soluble factors and BMSCs in certain cell lines, and impairs angiogenesis as well as osteoclast formation. Upregulation of *DKK1* suggests that the osteoblast suppressive effect of PTC-209 might be overcome by concurrent antibody treatment. Our data reveal therapeutic targeting of BMI-1 by PTC-209 as a promising novel therapeutic intervention for MM. Further studies examining the anti-myeloma activity of PTC-209 and more advanced BMI-1 inhibitors (e.g. PTC596) are therefore warranted.

## Methods

### Reagents

PTC-209, pomalidomide and carfilzomib were obtained from SelleckChem, dissolved in DMSO and stored at −80 °C. Dexamethasone was obtained from Sigma-Aldrich, dissolved in PBS and stored at −20 °C. Recombinant human IGF-1, IL-6, receptor activator of nuclear factor-kappa B ligand (RANKL) and macrophage colony-stimulating factor (M-CSF) were obtained from Peprotech, dissolved in PBS/BSA 0.1 % and stored at −20 °C. Goat anti-human DKK1 neutralizing antibody and normal goat IgG were purchased from R&D Systems, dissolved in PBS and stored at −20 °C.

### Cell lines and culture conditions

Human multiple myeloma cell lines (HMCLs) U266, KMS-12-BM, OPM-2, NCI-H929, SK-MM-1 and RPMI8226 were obtained from the German Collection of Microorganisms and Cell Cultures (Braunschweig, Germany). MM.1S and MM.1R cells were kindly provided by Dr. Steven Rosen (Northwestern University, Chicago, IL). A human bone marrow mesenchymal stromal cell line immortalized by enforced expression of telomerase (BMSC TERT^+^) was kindly provided by Dr. Dario Campana (St. Jude Children’s Research Hospital, Memphis, TN). All cell lines were cultivated in RPMI-1640 medium supplemented with 10 % heat-inactivated fetal bovine serum, 2 mM L-glutamine and 100 U/ml penicillin/streptomycin (Gibco). BMSC TERT^+^ cells were supplemented with 1 μM hydrocortisone (Sigma-Aldrich). For co-culture experiments, 1 × 10^5^ BMSC TERT^+^ cells were seeded in 24-well plates and cultured overnight before 2 × 10^5^ MM cells were added per well for 72 h.

### Cytotoxicity assay

Viability was determined by using Cell Counting Kit 8 (Sigma-Aldrich) following the manufacturer’s directions. In brief, HMCLs (2 × 10^4^), BMSC TERT^+^ cells (1 × 10^4^) and PBMCs (2.5 × 10^5^) were incubated in flat-bottomed 96-well plates (ThermoFisher Scientific) in the presence of PTC-209 (0.01–10 μM) alone, or in combination with either pomalidomide (1–5 μM) or carfilzomib (1–5 nM). Viability assessment in the presence of recombinant human IGF-1 (10 ng/ml) and IL-6 (10 ng/ml) was performed in serum-free Syn-H medium (ABCell-Bio). After 96 h, cells were incubated with WST-8, and absorbance was measured at 450 nm using a HTS 7000 Bio Assay Reader (Perkin Elmer).

### Colony formation assay

HMCLs (2 × 10^3^) either treated or untreated with PTC-209 at 1 μM were plated in duplicates in 1.1 ml methylcellulose-based medium (MethoCult Classic, StemCell Technologies) per 6-well and incubated for 14 days (37 °C, 5 % CO_2_). At the end of the incubation period, the number of colonies consisting of >40 cells was scored using an inverted microscope with ×4, ×10 and ×20 planar objectives.

### Flow cytometry

Induction of apoptosis was determined by Annexin V/7-AAD staining (BD Biosciences). HMCLs were seeded in the presence or absence of BMSC TERT^+^ cells and treated with 0.1 % DMSO (control), PTC-209 (1 μM), pomalidomide (1 μM) and/or carfilzomib (5 nM) for 72 h. Cells were incubated with Annexin V and 7-AAD for 15 min in the dark before performing analysis.

Cell cycle analysis was performed after treatment of HMCLs with PTC-209 (1 μM) for 24 h using the FxCycle™ PI/RNase Staining solution (ThermoFisher Scientific) following the manufacturer’s instructions.

Intracellular staining of BMI-1 and MCL-1 was performed using the BD Transcription Factor Buffer Set (BD Biosciences) according to the manual. After fixation and permeabilization, cells were incubated with a mouse anti-human MCL-1 antibody (ab197529, Abcam), mouse anti-human BMI-1 antibody (562528, BD Biosciences) or the corresponding isotype controls for 40 min at 4 °C. Thereafter, cells were washed and analysed. All analyses were performed on a FACScan (BD Biosciences).

### Quantitative RT-PCR

Total RNA was isolated using RNeasy kit (Qiagen), and cDNA synthesis was performed with M-MuLV reverse transcriptase (New England Biolabs). *CDKN1A*, *CDKN1B*, *MYC*, *CCND1*, *NOXA*, TRAP, cathepsin K and *DKK1* expression levels were analysed by quantitative PCR (qPCR) using TaqMan Universal PCR Master Mix and pre-designed TaqMan gene expression assays (Applied Biosystems). RPLPO served as endogenous control. Reactions were carried out in 25 μl volumes and run on the ABI Prism 7300 platform (Applied Biosystems). All samples were run at least in duplicates.

### PARP ELISA

Levels of cleaved PARP were analysed by using a commercially available ELISA kit (Invitrogen) following the manual. In brief, cell lysates were incubated with cleaved PARP detection antibody for 3 h at room temperature on an orbital shaker. Subsequently, wells were washed and incubated with anti-rabbit IgG HRP for 30 min at room temperature. After an additional wash step, 100 μl stabilized chromogen was added per well, the plate was incubated for 30 min at room temperature in the dark and finally mixed with 100 μl stop solution per well. Absorbance was measured at 450 nm, and levels of cleaved PARP were determined in relation to a standard curve.

### Osteogenic differentiation

BMSCs were seeded at a density of 25,000 per square centimetre and grown to 70–80 % confluence. Osteoblast differentiation was initiated by changing the medium to alpha-MEM supplemented with 15 % FBS, 2 mM L-glutamine, 100 U/ml penicillin, 100 μg/ml streptomycin, 10 nM dexamethasone, 50 μg/ml ascorbic acid and 5 mM β-glycerophosphate (Sigma-Aldrich). Osteogenic medium was changed every 3–4 days. PTC-209, normal goat IgG (1 μg/ml) and/or anti-DKK1 neutralizing antibody (1 μg/ml) were added with every medium change. Cells treated with 0.1 % DMSO served as control. Osteoblast formation was assessed by alkaline phosphatase activity assay and alizarin red S staining as described previously [53, 54].

### Osteoclast differentiation

Human PBMCs were obtained from voluntary healthy donors and cultured (2.5 × 10^6^/ml) overnight in 24-well plates to remove non-adherent cells. Subsequently, osteoclast differentiation was initiated by changing the medium to alpha-MEM supplemented with 10 % FBS, 2 mM L-glutamine, 100 U/ml penicillin, 100 μg/ml streptomycin, 50 ng/ml RANKL and 25 ng/ml M-CSF in the presence or absence of PTC-209. Medium was changed every 3–4 days. At day 14, cultures were fixed and stained using the Acid Phosphatase Leukocyte Kit (Sigma-Aldrich) according to the manufacturer’s instructions. Cells were evaluated using an inverted microscope and multinucleated TRAP-positive cells (>3 nuclei) were scored as mature osteoclasts.

### Tube formation

Tube formation was assessed following the manual of the Angiogenesis Starter Kit (Gibco). Briefly, human umbilical vein endothelial cells (HUVECs) were seeded in LVES-supplemented medium 200 at a density of 2.5 × 10^3^ cells per square centimetre. For in vitro tube formation, 80 % confluent cultures were harvested, and HUVECs (50,000 per 24-well plate) were plated on Geltrex® Matrix pre-coated wells in the presence or absence of PTC-209. Tube networks were documented 19 h post-seeding by using an inverted microscope. Analysis was performed using the Angiogenesis Analyzer for Image J (http://image.bio.methods.free.fr/ImageJ/?Angiogenesis-Analyzer-for-ImageJ.html).

### Gene expression analysis

For the illustration of *BMI-1* expression in CD138^+^ purified bone marrow samples as well as for the assessment of overall survival in different MM patients, following datasets from the Gene Expression Omnibus (GEO) were selected: GSE2658 (consisting of 559 samples from newly diagnosed MM patients treated within the TT2 and TT3 protocol), GSE5900 (22 healthy controls, 44 MGUS patients and 12 SMM patients), GSE6477 (15 healthy controls, 22 MGUS patients, 24 SMM patients, 73 newly diagnosed and 28 relapsed MM patients), GSE31161 (346 TT2 baseline and 127 TT2 relapse samples as well as 433 TT3 baseline and 29 TT3 relapse samples) and GSE9782 (264 relapsed and/or refractory MM patients treated with bortezomib or dexamethasone). The GSE9782 dataset was established by Mulligan et al. (2007) and contains data from 156 patients treated within the APEX phase 3 trial, 57 patients treated within a companion study to receive bortezomib after progressive disease with dexamethasone, 44 patients treated within the SUMMIT phase 2 trial and 7 patients treated within the CREST phase 2 trial [29].

Expression and clinical data were downloaded into R using the Bioconductor GEOquery package. For GSE6477, GSE5900 and GSE31161, raw CEL files were downloaded from GEO and analyses were performed on gcrma-normalized samples in R. In case of GSE2658, corresponding raw CEL files were downloaded from GEO study GSE24080 in order to perform a gcrma-normalization on the raw data using the ‘affy’ package from Bioconductor (since raw CEL files are not provided in the GSE2658 dataset). For GSE9782, mas5 expression sets were retrieved for analysis using the GEOquery package, since raw CEL files are not provided for this study.

### Statistical analysis

Survival analysis was performed in R using the ‘survival’ package. In order to split the patients into two groups with different survival probabilities exhibiting higher or lower *BMI-1* expression, the maximally selected rank statistics, implemented in the maxstat R package, was applied to the *BMI-1* expression data. The statistical significance of differences in overall survival between the two groups was calculated by the log-rank test, and survival curves were plotted using the Kaplan-Meier method. For the analysis of in vitro experiments, two-tailed unpaired *t* test was performed using Prism 5 (GraphPad Software Inc., La Jolla, CA, USA). *P* values <0.05 were considered to be statistically significant. Graphs represent the mean ± standard deviation of at least three independent experiments performed in triplicates. Synergistic, additive or antagonistic drug activities of combination treatments were determined by using CompuSyn software.

## Additional file

Additional file 1: PTC-209 displays additive and synergistic activity with dexamethasone. Additive/synergistic activity of drug combinations was confirmed by concurrent treatment of MM cell lines with PTC-209 and dexamethasone for 96 h at varying concentrations. Graphs for MM.1S and U266 are representative for the panel of HMCLs analysed. Combination index (CI) values were determined with CompuSyn. CI values <0.8, 0.8–1.2 or >1.2 indicate synergistic, additive or antagonistic drug activities, respectively. SK-MM-1 cells did not respond to dexamethasone at the concentrations used (viability >100 % at the end of the incubation period); determination of CI values was thus not possible. *NA* not applicable. (PDF 631 kb)

## Abbreviations

ALDH: aldehyde dehydrogenase
ALP: alkaline phosphatase
Bax: BCL2-associated X protein
Bim: BCL2-like 11
BM: bone marrow
BMI-1: polycomb complex protein BMI-1
BMSC: bone marrow stromal cell
CCND1: cyclin D1
CDKN1A: cyclin-dependent kinase inhibitor 1A
CDKN1B: cyclin-dependent kinase inhibitor 1B
CI: combination index
DKK1: Dickkopf-1
GEP: gene expression profiling
HDACi: histone deacetylase inhibitor
HMCL: human myeloma cell line
HUVEC: human umbilical vein endothelial cell
IGF-1: insulin-like growth factor 1
IL-6: interleukin 6
MCL-1: myeloid cell leukemia 1
M-CSF: macrophage colony-stimulating factor
MGUS: monoclonal gammopathy of undetermined significance
MM: multiple myeloma
MMSET: multiple myeloma SET domain
OS: overall survival
MYC: v-myc avian myelocytomatosis viral oncogene homolog
PARP: poly(ADP-ribose) polymerase
PBMC: peripheral blood mononuclear cell
PC: plasma cell
PRC1: polycomb repressive complex 1
RANKL: receptor activator of NF-kappa B ligand
SMM: smouldering multiple myeloma
TRAP: tartrate-resistant acid phosphatase
TT: total therapy
