# Supplementary material for: Targeting of BMI-1 with PTC-209 shows potent anti-myeloma activity and impairs the tumour microenvironment
Source: J Hematol Oncol. 2016 Mar 2;9:17. doi: 10.1186/s13045-016-0247-4 (PMC4776359; doi:10.1186/s13045-016-0247-4)
Supplement: Additional file 1: — PTC-209 displays additive and synergistic activity with dexamethasone. Additive/synergistic activity of drug combinations was confirmed by concurrent treatment of MM cell lines with PTC-209 and dexamethasone for 96 h at varying concentrations. Graphs for MM.1S and U266 are representative for the panel of HMCLs analysed. Combination index (CI) values were determined with CompuSyn. CI values <0.8, 0.8–1.2 or >1.2 indicate synergistic, additive or antagonistic drug activities, respectively. SK-MM-1 cells did not respond to dexamethasone at the concentrations used (viability >100 % at the end of the incubation period); determination of CI values was thus not possible. NA not applicable. (PDF 631 kb) [file 13045_2016_247_MOESM1_ESM.pdf]

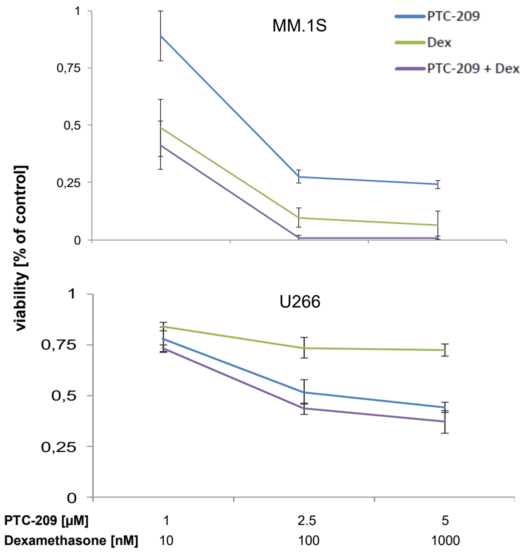

	PTC-209 [µM]	Dexamethasone [nM]	Combination Index
**KMS-12-BM**	1	10	1.3
	2.5	100	**0.50**
	5	1000	3.45
**OPM-2**	1	10	**0.24**
	2.5	100	**0.11**
	5	1000	**0.12**
**MM.1S**	1	10	1.27
	2.5	100	**0.10**
	5	1000	**0.19**
**NCI-H929**	1	10	**0.90**
	2.5	100	**0.24**
	5	1000	**0.59**
**SK-MM-1**	1	10	NA
	2.5	100	NA
	5	1000	NA
**U266**	1	10	**0.86**
	2.5	100	**0.57**
	5	1000	**0.84**
